# Maternal, dominance and additive genetic effects in Nile tilapia; influence on growth, fillet yield and body size traits

**DOI:** 10.1038/s41437-017-0046-x

**Published:** 2018-01-16

**Authors:** R Joshi, JA Woolliams, THE Meuwissen, HM Gjøen

**Affiliations:** 1 Department of Animal and Aquacultural Sciences, Norwegian University of Life Sciences, 1432 Ås, Norway; 2 The Roslin Institute, Royal (Dick) School of Veterinary Studies, University of Edinburgh, Easter Bush, Midlothian, EH25 9RG UK

## Abstract

There are only few studies of dominance effects in non-inbred aquaculture species, since commonly used mating designs often have low power to separate dominance, maternal and common environmental effects. Here, a factorial design with reciprocal cross, common rearing of eggs and subsequent lifecycle stages and pedigree assignment using DNA microsatellites was used to separate these effects and estimate dominance (d^2^) and maternal (m^2^) ratios in Nile tilapia for six commercial traits. The study included observations on 2524 offspring from 155 full-sib families. Substantial contributions of dominance were observed (*P* < 0.05) for body depth (BD) and body weight at harvest (BWH) with estimates of *d*^2^ = 0.27 (s.e. 0.09) and 0.23 (s.e. 0.09), respectively in the current breeding population. In addition the study found maternal variance (*P* < 0.05) for BD, BWH, body thickness and fillet weight explaining ~10% of the observed phenotypic variance. For fillet yield (FY) and body length (BL), no evidence was found for either maternal or dominance variance. For traits exhibiting maternal variance, including this effect in evaluations caused substantial re-ranking of selection candidates, but the impact of including dominance effects was notably less. Breeding schemes may benefit from utilising maternal variance in increasing accuracy of evaluations, reducing bias, and developing new lines, but the utilisation of the dominance variance may require further refinement of parameter estimates.

## Introduction

Genetic variation can be partitioned into additive and non-additive components of variance, where the latter arises from the interactions among loci (epistasis) or between alleles within a locus (dominance). Although sustained genetic change in conventional breeding schemes depends only on the additive component at the time of selection, the non-additive components can be utilised in the short-term through mate selection to obtain favourable heterosis in the offspring cohort, and in the long-term to protect the genetic assets of the breeder through F2-breakdown, e.g., through selection within lines or through selection schemes like Reciprocal Recurrent Selection (RRS) (Wei and Van der Steen 1991). In practice, commercial evaluations commonly use additive models ignoring the non-additive variation, but there is a continuing debate on whether the prediction accuracy is greater when models explicitly account for the non-additive genetic variation present (Wittenburg et al. 2011; Su et al. 2012; Muñoz et al. 2014).

Relatively few studies have investigated non-additive genetic effects in fish, compared to other animals, and these are limited to few species, especially salmon (Winkelman and Peterson 1994a, b; Rye and Mao 1998; Pante et al. 2002; Gallardo et al. 2010), trout (Vandeputte et al. 2002) and carp (Wang et al. 2006), possibly due to the demands of the design for estimation. These studies have mainly been done for weight traits only, where the dominance ratio (the fraction of phenotypic variances explained by dominance deviations) ranged from 0 to 0.62. Estimates of dominance variation are lacking in tilapia, though some studies have reported heterosis effects (Bentsen et al. 1998; Maluwa and Gjerde 2006a; Lozano et al. 2011).

It has been reported that the pedigree-based methods overestimate the dominance variation (Heidaritabar et al. 2016). For example, dominance and maternal effect may be confounded when analysing the data from hierarchical mating schemes (Mrode 2014); making it difficult to estimate the non-additive genetic effects precisely. In the present study, we have a factorial design with reciprocal cross, which is better suited to separate the maternal and non-additive genetic effects (Lynch and Walsh 1998; Shaw and Woolliams 1999; Vandeputte et al. 2004). The pedigree information further helps us to estimate the dominance variation by contrasting the parental dominance matrix from other effects attributed to the full-sib family groups.

The aim of this study was to study the magnitude of dominance variance, using a purpose-bred population of tilapia, on growth and morphological traits such as fillet yield. A further aim was to assess the impact on the genetic evaluation based on the effect on heritability and ranking of the selected animals.

## Materials and methods

### Experimental design

The data are from a trial conducted at Central Luzon State University (Munoz, Philippines) by GenoMar AS (Oslo, Norway) on Nile tilapia (*Oreochromis niloticus*) between 2014 and 2015. The test-groups studied were from the GST^*®*^ strain which originated from the well-documented GIFT strain (Bentsen et al. 2017). Pedigree was thus available all the way back to the population of crossbreds defined as the base of the GIFT breeding program, which was 17 generations before the formation of the test-groups.

**Fig. 1:**
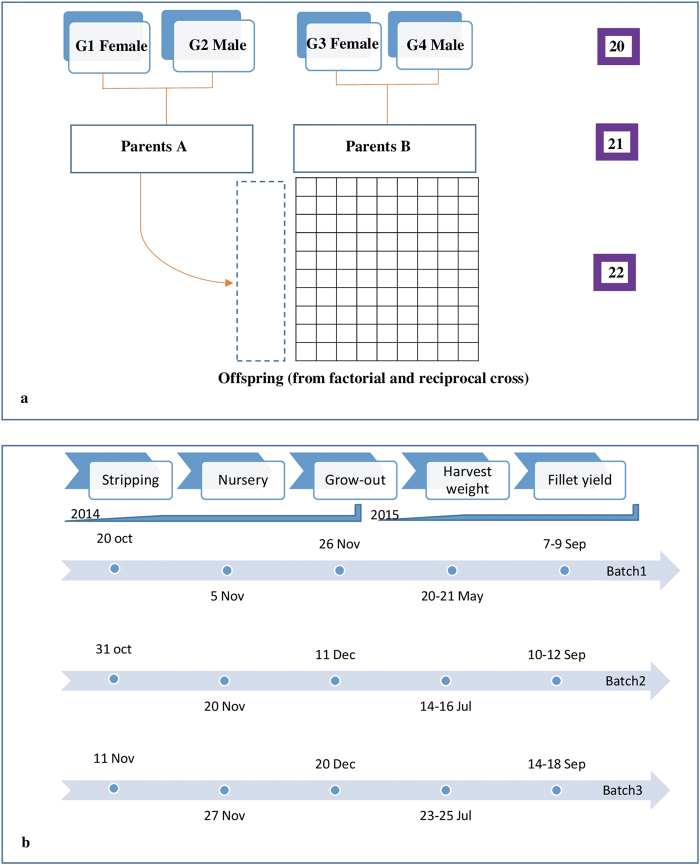
**a** The mating design used for the study. The numbers on right hand side represents the generation number of the GST^*®*^ strain. **b** Dates showing different phases of lifecycle of Tilapia. Offspring observed from the crosses of A and B were divided into three different batches and reared separately

The mating design for the study is shown in Fig. 1a. Males and females were chosen from four full-sib families (G1, G2, G3 and G4) in generation 20, with no parents in common. From these, two parent groups were created in generation 21: group A from a G1 × G2 cross, and group B from a G3 × G4 cross. The design was intended to have 1 female parent in each of G1 and G3, and 1 male parent in each of G2 and G4, however, the offspring of G1 were subsequently found to be from 2 females, although their offspring could not be distinguished by the genotyping procedures described later. Within parent groups, 10 males and 11 females were selected from group A and 10 males and 13 females from group B. From these, A × B and B × A crosses were produced with full factorial matings across parent groups, i.e., all A females were mated with all B males, and all B females were mated with all A males. From each of these full-sib families, in Generation 22, offspring were chosen at random for rearing.

### Rearing procedure

The offspring were all produced by artificial fertilisation, i.e., stripping, in three batches, which were reared separately, following the schedule of Fig. 1b. Eggs stripped from the genital papilla of ready to spawn females were fertilised in mixing containers by stripping milt from male (eggs stripped from one female was divided equally among males at 80 eggs per pool) in the wet lab and immediately transferred to incubators. There was no mouth brooding, which is common in Tilapia. To reduce the common environmental effect, the families were kept and reared together once the eggs hatched or the yolks were completely absorbed, whereas in most conventional schemes, using physical tags, families need to be kept separate until they can be tagged at a size of ca 15 g, i.e., for another 5–7 weeks. The fishes were stocked in fine-mesh nursery cages at rates of 143, 157, and 149 individuals/m^2^ for batches 1–3, respectively, with corresponding survival rates of 85, 95, and 86%. All offspring were hormonally treated, so were either males or sex-reversed males, which is normal aquacultural procedure. After 21 days, tilapias were transferred to earthen grow-out ponds with stocking rate of 1.3, 1.4, and 1.5 individuals/m^2^ for batches 1–3, respectively. The fish was reared under semi-intensive condition, with green-water management supplemented with commercial feed as per Genomar standard protocol (Table S1.2 and S1.3 (Supplementary 1)).

### Harvesting

A total of 2987 offspring were collected after 6–7 months in the grow-out ponds, and were held or stored by batches in net cages prior to filleting, as shown in Fig. 1b. The fishes were collected smaller than normal commercial fileting size due to expected typhoon season. At collection, records were obtained for body weight (BWH), body depth (BD) and body length (BL) (Figure S1.1 (Supplementary 1)). At filleting, records of body weight (BW), body thickness (BT) and Fillet weight (FW) were obtained. Fillet yield (FY) was calculated as the ratio between fillet weight (FW) and body weight at filleting (BW) and expressed as percentage. Days of collection and filleting are shown in Fig. 1b. Batches 1 and 2 were filleted by the same three filleters, whereas batch 3 was filleted by only two of them. The data are presented in Supplementary 5.

### Pedigree

Parental assignment was done by inference from 9 microsatellite markers, using DNA obtained from fin clips for parental groups A and B, and all their offspring at Temasek Life Sciences, Singapore. These microsatellites were selected from several hundred markers available, e.g., Kocher et al. (1998) and Lee et al. (2005), and the 9 markers chosen were all highly variable and could be run in multiplex, i.e., 9 markers in a single PCR run. The parental assignment was based on the mendelian exclusion, which is on number of hits (synonymous markers) between parent groups and offspring. It was, to minimum extent, allowed for missing genotypes or genotyping error, and the offspring having highest hit with a certain parent gets assigned to this parent (Woo-Jai Lee, personal communication).

Parentage could not be assigned for 15.6% individuals, leaving records from a total of 2524 individuals; 1318 from A × B and 1206 from B × A. These offspring were from 155 full-sib families with an average of 16.3 offspring per full-sib family (SD = 12.3, range: 1–59). The main reason for the low assignment rate is that the marker set do not have enough exclusion power for the family structure used in the cross-breeding scheme, which involved only more closely related breeders. Because of the factorial mating design, we had a lot of half-sib families, which made it harder to uniquely assign individuals. Therefore, some fish would fit equally well into 2 or more families. With no way of knowing which family was the correct one, these were set as unassigned. The complete distribution of offspring across parents and families is given in Table S1.1 (Supplementary 1).

The established pedigree from generations 3 to 22 contained 4051 records (Supplementary 6), and its structure and depth is shown graphically in Figure S1.2 (Supplementary 1). The mean inbreeding level over generations 9–18 with a mean value of 0.061 for G1–G4 is shown graphically in Figure S1.3 (Supplementary 1); being 0.061 in generation 20. The estimate of effective population size calculated using the pedigree information from generations 9 to 18 was 95 (See Figure S1.4- Supplementary 1).

### Statistical analysis

ASReml-4 (Gilmour and Thompson 2014) was used to fit mixed linear models, using REML to estimate variance components and breeding values for the six traits described above. A model with additive, dominance and maternal effects (ADM) was the full model used for analysis (see below) with dominance, maternal or both effects removed to test for their significance: sub-models AD was fitted omitting maternal effect, AM was fitted omitting dominance effect and A was fitted omitting both dominance and maternal effects. The ADM model was

$${\rm{ADM\, Model}}:\quad \quad \quad {\mathbf{y}} = {\mathbf{Xb}} + {\mathbf{Z}}_{\mathrm{1}}{\mathbf{a}} + {\mathbf{Z}}_{\mathrm{2}}{\mathbf{d}} + {\mathbf{Z}}_{\mathrm{3}}{\mathbf{m}} + {\mathbf{e}},$$

where **y** is the vector of records; **b** is the vector of fixed effects, which were type of reciprocal cross (1 d.f.) and other systematic effects such as batch (2 d.f.) and day of collection (7 d.f.) or filleting (as appropriate, 10 d.f.); **a** is a vector of random additive genetic effects; **d** is vector of random dominance effects; **m** is vector of maternal effects; **e** is a vector of random residual errors and **X**, **Z**_1_, **Z**_2_ and **Z**_3_, are corresponding design matrices for fixed and random effects. For FW and FY, the fixed model also included filleter (2 d.f.)

Vectors **a** and **d** had effects for each individual in the pedigree; **m** for each full-sib family and **e** for each offspring. Their distributional assumptions were multivariate normal, with mean zero and

$${\rm{Var}}\left[ \begin{array}{l}{\mathbf{a}}\\ {\mathbf{d}}\\ {\mathbf{m}}\\ {\mathbf{e}}\end{array} \right] = \left[ {\begin{array}{*{20}{c}} {{\mathbf{A}}\sigma {\rm{_A}}^2} & 0 & 0 & 0 \\ 0 & {{\mathbf{D}}\sigma {\rm{_D}}^2} & 0 & 0 \\ 0 & 0 & {{\mathbf{I}}\sigma {\rm{_M}}^2} & 0 \\ 0 & 0 & 0 & {{\mathbf{I}}\sigma {\rm{_E}}^2} \end{array}} \right],$$

where *σ*^2^_A_, σ^2^_D,_ *σ*^2^_M_ and *σ*^2^_E_ are additive genetic variance, dominance genetic variance, maternal variance and error variance, respectively; **A** is the numerator relationship matrix derived from pedigree; **D** is the matrix of coefficients of fraternity for individuals in the pedigree; and **I** is an identity matrix of appropriate size. The phenotypic variance was calculated as *σ*^2^_P_ = *σ*^2^_A_ + *σ*^2^_D_ + *σ*^2^_M_ + *σ*^2^_E_.

The estimated variance components were expressed relative to the total phenotypic variance (*σ*^2^_P_): additive heritability (*h*^2^) = *σ*^2^_A_ / *σ*^2^_P_, dominance ratio (*d*^2^) = *σ*^2^_D_ / *σ*^2^_P_, maternal ratio (*m*^2^) = *σ*^2^_M_ / *σ*^2^_P_. Goodness of fit was tested using likelihood ratio tests. The critical values for testing *H*_0_: *σ*^2^ = 0 against an alternative *H*_1_: *σ*^2^ > 0 with type 1 error of 0.05 was taken from the 90 percentile of *χ*_1_^2^, i.e., 2.71.

The coefficient of fraternity between individuals *x* and *y* (∆_*xy*_) was calculated following Lynch and Walsh (1998):

$${\rm{\Delta}} _{xy} = \frac{{{\mathbf{A}}_{ik} \times {\mathbf{A}}_{jl} + {\mathbf{A}}_{il} \times {\mathbf{A}}_{jk}}}{{\rm{4}}}\,{\rm{for}}\,x \ne y,$$

where *i* and *j* represents the sire and dam of *x*, *k* and *l* represents the sire and dam of *y*, **A**_*xy*_ is the numerator relationship between the individuals as shown in the subscripts and *F* is the inbreeding coefficient. For *x* = *y*, the coefficients were scaled by (1–*F*) to incorporate corrections for inbreeding as per Harris (1964). The scatterplot and density plots for **A** and **D** matrix for all the individuals in the pedigree and for the phenotyped individuals are shown in Figure S1.5 (Supplementary 1). To fit the models, the inverse of **D** is required and this was calculated using the R package ‘nadiv’ (Wolak 2012).

Variations on this ADM model were also investigated. Firstly, the pedigree was reduced to 3 generations, treating Generation 20 as the base generation so that the estimates of *h*^2^, *m*^2^ and *d*^2^ correspond more closely to a randomly mated cohort of the current population rather than the GIFT base. These were designated as A*D*M* models and procedures were identical to the ADM models other than the definition of the pedigree base.

Secondly, the analyses were conducted with a simple diallel model used to decompose the variances, which were designated SFM models (model with sire, full-sib family and maternal effects).

$$SFM\,{\mathrm{model}}\quad \quad \quad {\bf{y}} = {\bf{Xb}} + {\bf{Z}}_{\rm{4}}{\bf{s}} + {\bf{Z}}_{\rm{5}}{\bf{m}} + {\bf{Z}}_{\rm{6}}{\bf{f}} + {\bf{e}}$$

$${\rm{Var}}\left[ \begin{array}{l}{\mathbf{s}}\\ {\mathbf{m}}\\ {\mathbf{f}}\\ {\mathbf{e}}\end{array} \right] = \left[ {\begin{array}{*{20}{c}} {{\mathbf{I}}V_{{\rm{Sire}}}} & 0 & 0 & 0 \\ 0 & {{\mathbf{I}}V_{{\rm{Dam}}}} & 0 & 0 \\ 0 & 0 & {{\mathbf{I}}V_{{\rm{Fsib}}}} & 0 \\ 0 & 0 & 0 & {{\mathbf{I}}V_{\rm{E}}} \end{array}} \right],$$

where, the fixed effects **b** and design matrix **X** were as described for ADM models; **s** is a vector of random sire effects; **m** is a vector of random dam effects; **f** is the vector of full sib family effects; **Z**_4_**, Z**_5_ and **Z**_6_ are the design matrices corresponding to sire, dam and full-sib family effects. The variances attributable to the sire and dam, *V*_Sire_ and *V*_Dam_ were constrained to be equal in models S and SF models (appropriate for additive genetic contributions), with *V*_Fsib_ constrained to be 0 in S and unconstrained in SF. Model SM and SMF had V_Sire_ and *V*_Dam_ unconstrained with V_Fsib_ constrained to be 0 in SM and unconstrained in SFM. The phenotypic variance was estimated as *V*_P_ = *V*_Sire_ + *V*_Dam_ + *V*_Fsib._ Heritabilities, maternal and dominance ratio were estimated as *h*^2^ = 4*V*_Sire_ / *V*_P_ and *d*^2^ = 4*V*_Fsib_ / *V*_P_ and *m*^2^ = (*V*_Dam_ − *V*_Sire_) / *V*_P_.

Effects on the genetic evaluation was compared among the different models; by Pearson’s correlation between estimates of breeding values, ranking of the 100 best offspring (animals with phenotypes) and then counting the numbers that would have been excluded from the selected group compared to the simple A model.

## Results

### Descriptive statistics

Descriptive statistics for the six different traits are shown in Table 1. The coefficient of variation (CV) among traits ranged from 10% for body sizes (BD, BL, BT) and FY to >30% for BWH and FW.

**Table 1:** Descriptive statistics for BD (cm), BL (cm), BT (mm), BWH (g), FW (g) and FY (%), where N is the number of observation, SD and SE are standard deviation and error respectively and CV is the coefficient of variation expressed as %

Traits	N	Min	Max	Median	Mean (SE)	SD	CV (%)
BD	2524	5.00	12.00	8.70	8.86 (0.02)	1.00	11
BL	2524	14.10	28.20	22.40	22.37 (0.04)	2.14	10
BT	2513	23.50	59.70	40.40	40.65 (0.09)	4.40	11
BWH	2524	107.80	804.70	385.70	403.83 (2.48)	124.82	31
FW	2524	16.20	342.60	134.50	141.51 (1.02)	51.37	36
FY	2513	12.12	54.67	33.01	32.64 (0.06)	3.19	10

### Reciprocal cross effects

Numerical differences between reciprocal cross means were not statistically significant, although B × A were observed to have greater sizes and weights and FY; ranging from 0.1% for FY to 0.4% for BWH.

### Goodness of fit

The outcomes of the likelihood ratio test for goodness of fit are presented in Table 2. The traits could be separated into three distinct groups: BL and FY showed no evidence of maternal and dominance effects; BT and FW showed evidence of maternal effects only; whereas BWH and BD showed evidence of significant maternal and dominance effects. There was direct correspondence in the significance of these sources of variation (dominance and maternal) across the classes of model ADM, A*D*M* and SFM. This is explained in Supplementary 2.

**Table 2:** Log likelihood values of different models for the six traits

Model	BD	BL	BT	BWH	FW	FY
*ADM models*						
A	−81.786	−90.60	−56.94	−28.34	−21.95	−68.37
AD	−79.494^+^	−90.34	−56.14	−26.66^+^	−21.46	−68.37
AM	−78.220^+^	−89.38	−55.55^+^	−24.15^+^	−15.59^+^	−68.33
ADM	−75.660^↑^	−89.05	−54.57	−22.19^↑^	−14.80	−68.33
A*D*M* models						
A*	−81.79	−90.61	−56.94	−28.33	−21.94	−68.36
A*D*	−79.50^+^	−90.36	−56.14	−26.66^+^	−21.45	−68.36
A*M*	−78.23^+^	−89.38	−55.55^+^	−24.15^+^	−15.60^+^	−68.32
A*D*M*	−75.65^↑^	−89.05	−54.58	−22.18^↑^	−14.83	−68.32
*SFM models*						
S	−81.787	−90.61	−56.94	−28.34	−21.95	−68.37
SF	−79.498^+^	−90.36	−56.14	−26.66^+^	−21.45	−68.37
SM	−78.225^+^	−89.38	−55.55^+^	−24.15^+^	−15.59^+^	−68.33
SFM	−75.645^-^	−89.05	−54.57^↑^	−22.17^−^	−14.83^−^	−68.33

In animal models, superscripts +, − and ↑ are used to denote significance tests (LRT) within the hierarchy of models. Superscript + indicates significance over model A, and ↑ indicates significance over A, AD and AM models. Similarly, in Sire and Dam models, + indicates significance over model S, ↑ indicates significance over S and SF models, and − indicates significance over S, SF and SM models

### Estimates of heritabilities

**Fig. 2:**
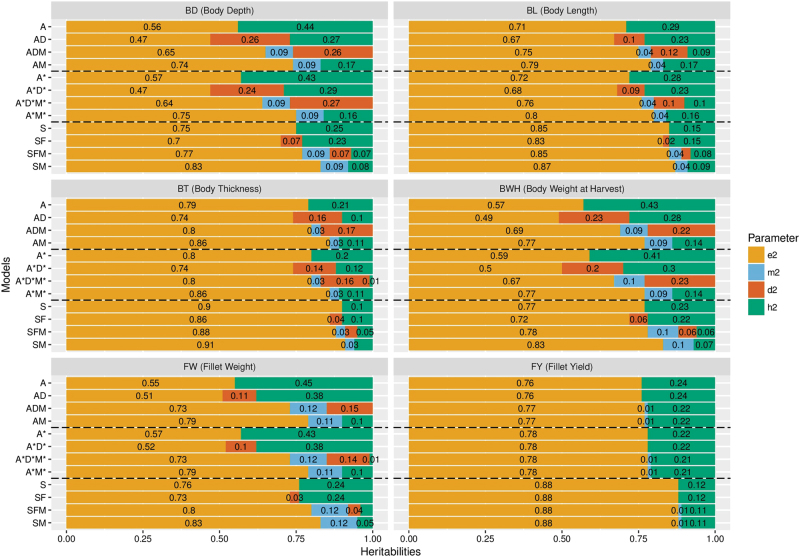
Decomposition of phenotypic variance into additive (*h*^2^), dominance (*d*^2^), maternal (*m*^2^) and residual (*e*^2^) components for the six traits studied. Missing values of *m*^2^ for some model means that the values are similar to the values obtained from other models for same trait. A was fixed to zero in the ADM model for all traits except BL, D was fixed to zero in both the AD and the ADM models for the trait FY, and *F* was fixed to zero or was in borderline in the SFM and SF models for FY

Estimates for the variance components and heritabilities for different traits obtained by the different models are shown graphically in Fig. 2 and in detail in Table S3.1 (Supplementary 3). The summary of the models of best fit for all the traits are given in Table 3.

**Table 3:** Heritabilities and phenotypic variances for the models of best fit for different traits (SE are in parentheses)

Traits	SFM models				A*D*M* models			ADM models		
	*h* ^2^	*d* ^2^	*m* ^2^	*σ* ^2^_P_	*h* ^2^	*d* ^2^	*m* ^2^	*h* ^2^	*d* ^2^	*m* ^2^
BD	0.07 (0.04)	0.07 (0.04)	0.09 (0.04)	0.51 (0.03)	0 (0)	0.27 (0.09)	0.09 (0.04)	0 (0)	0.26 (0.09)	0.09 (0.04)
BWH	0.06 (0.03)	0.06 (0.04)	0.10 (0.04)	6681 (355)	0 (0)	0.23 (0.09)	0.10 (0.04)	0 (0)	0.22 (0.08)	0.09 (0.04)
BT	0.06 (0.03)	—	0.03 (0.02)	8.89 (0.31)	0.11 (0.06)	—	0.03 (0.02)	0.11 (0.06)	—	0.03 (0.02)
FW	0.05 (0.03)	—	0.12 (0.05)	1002 (58)	0.10 (0.05)	—	0.11 (0.05)	0.10 (0.05)	—	0.11 (0.05)
BL	0.15 (0.05)	—	—	3.00 (0.11)	0.28 (0.08)	—	—	0.29 (0.08)	—	—
FY	0.12 (0.04)	—	—	8.95 (0.29)	0.23 (0.07)	—	—	0.24 (0.07)	—	—

The simple models gave the greatest additive genetic variances, and greatest h^2^ for all traits. The inclusion of dominance in the models decreased the additive variance in ADM and A*D*M* models but only marginally in SFM models. In contrast, including maternal effect decreased the additive genetic variance considerably for some traits. ADM and A*D*M* models gave similar results for all the traits.

**Fig. 3:**
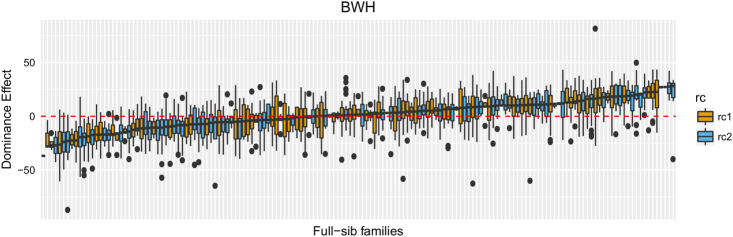
Figure showing the boxplots of the dominance deviations for individuals in different full-sib families obtained from ADM models for BWH (g). Boxplots are colour coded for the reciprocal crosses

For BWH and BD, the two traits for which the best fit included dominance, the dominance ratio was found to be 0.06 ± 0.04 and 0.07 ± 0.04 using the SFM model, but was much greater, with corresponding greater standard errors, for ADM and A*D*M* models; 0.27 ± 0.09 and 0.23 ± 0.08, respectively for the A*D*M* models. The dominance deviation among and within the different full-sib families for BWH are presented in Fig. 3, indicating large differences in expressed dominance effects.

For the four traits where evidence of maternal ratio (*m*^2^) was found (*P* < 0.05), the fraction was close to 0.1 for FW, BD and BWH; but was smaller for BT.

As shown in Fig. 2, *h*^2^ for all traits other than FY depend heavily on the model fitted. For best fit A*D*M* models, the estimates of h^2^ were moderate for BL and FY (0.28 and 0.23, respectively) which showed only additive variation; small for BT and FW (0.11 and 0.1, respectively), where there was evidence of maternal effects but no dominance, and 0 for BD and BWH, which showed both dominance and maternal variation. In the latter case, estimates of *h*^2^ from SFM models were small (0.07 ± 0.04 and 0.06 ±/ 0.03 for BD and BWH, respectively) rather than 0.

### Change in ranking

The difference in ranking of Estimated Breeding Values (EBVs) among the 100 best animals, as a result of different models and the use of different depth of pedigree, is presented in Table 4, for which the cohort using the simple A model has been used as a reference group for each trait. Adding only dominance effect made only minor differences in the top 100 list, with only 1–6% of the animals changing across the various traits. In contrast including maternal effect changed ~50% of the animals in the list for traits where best fit models indicated maternal variance, with much smaller impacts for BT and FY, where the maternal effect was not statistically significant. There was very little difference between ADM and A*D*M* models, showing the change of base from generation 3 to 20 had little impact. SFM models are not shown in Table 4 as these do not provide estimates of EBV.

**Table 4:** The impact of model choice for the top 100 animals after ranking animals on EBVs for the six traits compared to a model fitting only additive genetic variance or an A model

Models	Comparison based on A model					
	BD	BWH	BT	FW	BL	FY
A	0	0	0	0	0	0
AD	6	1	4	1	1	0
ADM	—	—	—	—	48	5
AM	52	52	26	53	43	5
A*	0	0	0	0	0	0
A*D*	7	2	4	1	1	0
A*D*M*	—	—	30	58	48	5
A*M*	52	52	26	53	43	5

The number shown is the number of top animals in A models that are excluded when fitting an alternative model, therefore the 0 for the A model is by definition. The dash indicates no additive variation was detected and so no EBVs were available

### Correlation of the EBVs

**Fig. 4:**
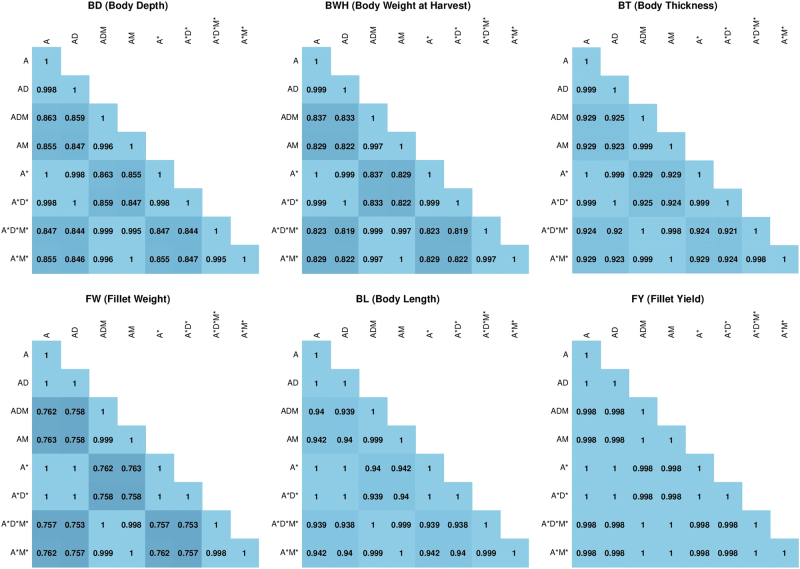
Correlation values of the EBVs for different models and traits (The colour has been coded from dark to light blue, signifying low correlation for darker colours). Please note: A was fixed to zero in the ADM model for all traits except BL, D was fixed to zero in both the AD and the ADM models for the trait FY, and F was fixed to zero or was in borderline in the SFM and SF models for FY

The correlation of the EBVs for all animals with observations among the different models are presented in Fig. 4. The correlations were close to 1 when only dominance term was added, i.e., changing from A to AD or AM to ADM or the analogous changes in A*D*M’ models, which is consistent with the outcomes of the ranking shown in Table 4.

However, including the maternal effects was found to be different for different traits, ranging from 0.76 for FW to 0.94 for BL. For FY, the correlation was 1, as the maternal effect was close to 0. As with the ranking, there was little impact from changing the base of generation 3 (ADM models) to generation 20 (A*D*M* models)

## Discussion

To the best of our knowledge, these are the first published results on dominance ratios in tilapia, and are potentially important for commercial production, both for the accuracy of EBVs for use in selection and for the eventual utilisation of heterotic effects. These were obtained by separating out the additive and non-additive genetic effects from the maternal and common environmental effects. This was achieved using a factorial mating design, including reciprocal crosses, and exploiting the large full-sib family sizes possible in fish species, which is uncommon in livestock, and seldom used in commercial aquaculture. The scope of the trial encompassed both the commercially important morphological and weight-related traits, and the post-harvest measures of fillet weight and yield, which characterise the primary saleable product.

The GST^®^ strain used for this study is derived from the GIFT strain that is the common ancestor to most tilapia populations used for commercial breeding, and the first 10 generations of GST^®^ also correspond to the first 10 generations of the GIFT strain. The designated base generation of GIFT, which here is defined as generation 3 of GST^®^, was formed from four wild and four Asian strains crossed systematically over 3 generations to allow mixing of the strains before selection for growth was commenced (Eknath et al. 1993). This origin from several diverse strains would prompt a hypothesis that there may have been substantial non-additive genetic variation in this base. The heterosis between different pairs of founding strains was reported range from <1 to 14% for BWH (Bentsen et al. 1998). For *Oreochromis shiranus*, a different tilapia species, the heterosis between strains in F1 crosses was up to 15% for BWH (Maluwa and Gjerde 2006b). The continued existence and the magnitude of the initial non-additive variation in the current GST^*®*^ strain would be subject to the changes in the frequencies of alleles underlying this variation, and the partition between dominance and additive variation will change over time accordingly (Falconer et al. 1996). Estimation of the base variances using the ADM linear mixed models does not account for these changes in allele frequency.

### Source of dominance variation

This variance parameters obtained from the ADM, A*D*M* and SFM models are all interpretations of the same three core variance components that are intrinsic to the factorial design, as shown in Supplementary 2. These core components are the variances among sires (*V*_Sire_), variances among dams (*V*_Dam_), and the variances within full-sib families (*V*_Fsib_). The supplementary information (Supplementary 4) shows that projecting *V*_Fsib_ to estimate *σ*^2^_D_ in the GST^®^ base generation results in a 4.5-fold scaling of the value that would be obtained from a standard assumption that *V*_Fsib_ is ¼*σ*_D_^2^. This explains why a small variance component in SFM models can translate into substantial estimates of *σ*^2^_D_ in ADM models. Furthermore, estimates of *σ*^2^_A_ from V_Sire_ are influenced by the design in that the sires used within parent groups A and B are full sibs. Therefore, the models produce a range of estimates that might be considered: empirical SFM estimates assuming *σ*^2^_A_ = 4*V*_Sire_, *σ*^2^_M_ = (*V*_Dam_ – *V*_Sire_), if >0; and *σ*^2^_D_ = 4*V*_Fsib_; A*D*M* estimates with a base generation in generation 20, which most closely correspond to random mating in the current population; and ADM estimates which project back to generation 3, the GIFT base. Since each emerge as scaling of the same set of core components, the standard errors and uncertainties reflect the magnitude of the scaling factors applied. The near-equal scaling factors from using generation 3 (ADM) or 20 (A*D*M*) as the base, demonstrate that the scaling observed for estimates of *σ*^2^_D_ in ADM models is a consequence of the design rather than the additional pedigree. There are additional approximations in the use of the fraternity matrix to assess dominance, as it is an approximation of the full dominance model (for example, Shaw and Woolliams (1999)), and it excludes terms that increase in importance with the inbreeding coefficient, F. The relatively low value of F suggests this may not be a serious problem in ADM models, and for A*D*M models with a generation 20 base, where *F* = 0.

### Estimates of different variance components

It has been assumed that *V*_Fsib_ can be interpreted as dominance variance, an assumption common to many other studies. Although our design has separated out the maternal effect and minimised common environmental effects through the management described in the Materials and Methods, this interpretation cannot be certain. The results show that maternal variance is still detectable for four of the six traits (not for BL and FY) despite this management. These effects might be related to the size and quality of the eggs or mitochondrial effects. Large eggs have more yolk reserves and have been shown to be positively correlated to the growth and development of fry (Rana 1985; Springate and Bromage 1985). There has been no separate reporting of maternal ratio in the tilapia studies listed in Table S3.2 of Supplementary 3, since their design did not allow to separate them from common environment or full-sib family effects (e.g. GIFT has a hierarchical mating design).

The estimated *h*^2^ for all traits, except for BWH and FW, are within the ranges of those published for GIFT (Table S3.1 of Supplementary 3) although for BWH and FW our estimates are towards the low end of the range. One contributing reason for this is that we have used the complex models which will have removed maternal and full-sib variances that may have been miss-attributed in simpler models. For example, the best-fit estimate of *h*^2^ for BWH, which tends to be particularly low in comparison to other estimates from GIFT or GST^®^, is 0.40 for the A* model, which is similar to the other published estimates for GIFT and GST^®^. However, the low heritability estimates reported in the present study, must be evaluated as too low, since the realised genetic gain found in many tilapia studies, e.g., as reported by Bentsen et al. (2017). On the other hand, such high selection response is expected in the initial phase of a breeding program, since considerable “Bulmer effect” will cause higher selection response than in later phases of the selection program (Bulmer 1971). The correct heritability estimates thus probably will be somewhere between these boundaries.

There have been no previous estimates of dominance ratios in tilapia, but very few in other fish species, including the more intensively studied trout and salmon (Winkelman and Peterson 1994a, b; Rye and Mao 1998; Pante et al. 2002; Gallardo et al. 2010), with moderate values of the dominance ratio for BWH. But the comparison is not straightforward, the mating designs in these studies have low power to separate the dominance and common environment effect; the source for the dominance variation being only from the multi-generational pedigree, with phenotypes available at each generation. The significance and the standard errors of the *d*^2^ were not reported for Atlantic salmon (Rye and Mao 1998); and *d*^2^ was not significantly different from zero for chinook salmon (Winkelman and Peterson 1994a, b). Although *d*^2^ was stated as significant (0.19 and 0.06 for two populations, but with no s.e.) for coho salmon (Gallardo et al. 2010), they were unable to separate dominance from common environment precisely with their applied mating and rearing design.

### Implications for aquaculture production

The maternal component, shown to be present in all but two of the traits, has practical consequences for the genetic evaluation. This source of variance is not always fitted, however, as shown in Table 4, it can have substantial consequences on the rankings of selection candidates. Furthermore, ignoring the term will tend to inflate the heritability and consequently introduce bias into evaluations; over-predicting potential gains. It also places importance on management steps to minimise the size of this component, although, as yet it may not be feasible to remove the component completely from all traits, as demonstrated in this study. The finding that some traits exhibit dominance variance will likely require further research as its magnitude remains uncertain and obtaining further information remains challenging, although genomics may offer new opportunities because high-density SNP genotypes provide more individual genomic information, potentially leading to more accurate estimate of the relationships and dominance variance (Vitezica et al. 2013; Heidaritabar et al. 2016). Including dominance, terms when parameters are open to substantial error may reduce the accuracy of prediction rather than improve it (Sales and Hill 1976). Furthermore, adding dominance to models had little impact on ranking the EBVs in this study and have had only marginal benefit in other sectors (e.g. Sun et al. 2014). However, the findings do open consideration of specialised breeding options. The maternal variance may be heritable, and instead of minimising it there may be opportunities for breeding specialised maternal and sire lines to breed crossbred fish using reciprocal recurrent selection, which could become more attractive if further research confirms the existence of substantial dominance variance in commercially important traits. This may also involve the utilisation of the relatively large differences in expressed dominance effects among and within families, as shown in Fig. 3.

### Data Archiving

The phenotypic data and the pedigree are available as Supplementary 5 and 6 respectively.

## Electronic supplementary material

Supplementary 1 Design of the study

Supplementary 2 Correspondence between SFM, ADM and A*D*M* models

Supplementary 3 Tables of variance component estimates for all the models

Supplementary 4 Literature reviews for heritabilities and common maternal and environmental effects in tilapia

Supplementary 5 - Phenotypic data

Supplementary 6 - Pedigree data
