# Supplementary material for: Maternal, dominance and additive genetic effects in Nile tilapia; influence on growth, fillet yield and body size traits
Source: Heredity (Edinb). 2018 Jan 16;120(5):452–62. doi: 10.1038/s41437-017-0046-x (PMC5889400; doi:10.1038/s41437-017-0046-x)
Supplement: Supplementary file 1 — Supplementary 1 Design of the study [file 41437_2017_46_MOESM1_ESM.pdf]

Supplement Information 1

Maternal, dominance and additive genetic effects in Nile tilapia; influence on growth, fillet yield and body size traits

R Joshi[1], J Woolliams[1,2], THE Meuwissen[1] and HM Gjøen[1]

[1]Department of Animal and Aquacultural Sciences, Norwegian University of Life Sciences, 1432 Ås, Norway

[2] The Roslin Institute, Royal (Dick) School of Veterinary Studies, The University of Edinburgh, Easter Bush Campus, Midlothian, Scotland, United Kingdom

Design of the study

Table S1.1: Observations in each factorial mating. 18 different sires and dams are mated in factorial manner

A x B	S1	S2	S3	S4	S5	S6	S7	S8	S9	Total
**D1**	5	-	3	5	1	4	2	-	-	**20**
**D2**	9	-	6	3	2	2	6	4	-	**32**
**D3**	30	6	46	24	13	36	14	17	14	**200**
**D4**	5	8	8	9	2	4	-	4	6	**46**
**D5**	5	7	4	3	1	3	-	6	3	**32**
**D6**	26	20	39	13	8	10	16	17	12	**161**
**D7**	26	12	44	13	13	30	13	17	22	**190**
**D8**	34	25	59	19	22	47	15	34	15	**270**
**D9**	15	10	17	8	8	10	12	7	8	**95**
**D10**	35	27	54	16	21	52	45	4	18	**272**
**Total**	**190**	**115**	**280**	**113**	**91**	**198**	**123**	**110**	**98**	**1318**

B x A	S10	S11	S12	S13	S14	S15	S16	S17	S18	Total
**D11**	30	29	13	32	29	35	19	16	22	**225**
**D12**	20	18	7	9	16	27	3	15	13	**128**
**D13**	18	25	19	16	26	16	14	22	17	**173**
**D14**	11	6	6	8	9	8	3	6	4	**61**
**D15**	33	36	23	32	37	38	19	28	26	**272**
**D16**	9	8	11	7	11	17	7	12	15	**97**
**D17**	3	13	6	2	3	6	1	4	3	**41**
**D18**	16	36	14	30	27	30	10	29	17	**209**
**Total**	**140**	**171**	**99**	**136**	**158**	**177**	**76**	**132**	**117**	**1206**

Table S1.2: Minimum and maximum temperature during tilapia production phase

**Month**	**Temperature (ºC)**	
	**Min**	**Max**
Nov. 2014	28	31
Dec. 2014 (1-15)*	27	30
Dec. 2014 (16-31)*	25	29
Jan. 2015	25	28
Feb. 2015	25	28
Mar. 2015	27	30
Apr. 2015	27	31
Aug. 2015	28	31
Sep. 2015	30	32

*Numbers inside the parenthesis indicate days.

Table S1.3: Types of commercial feed fed during different tilapia production stages

**Size of fish**	**Type**
AI/fry	Booster1
Nursery	Booster1 & 2
1-10 g	Booster3
11-20 g	Pre-starter
20-80 g	Starter
81 g & above	Grower
Breeders	Broodstock/Grower

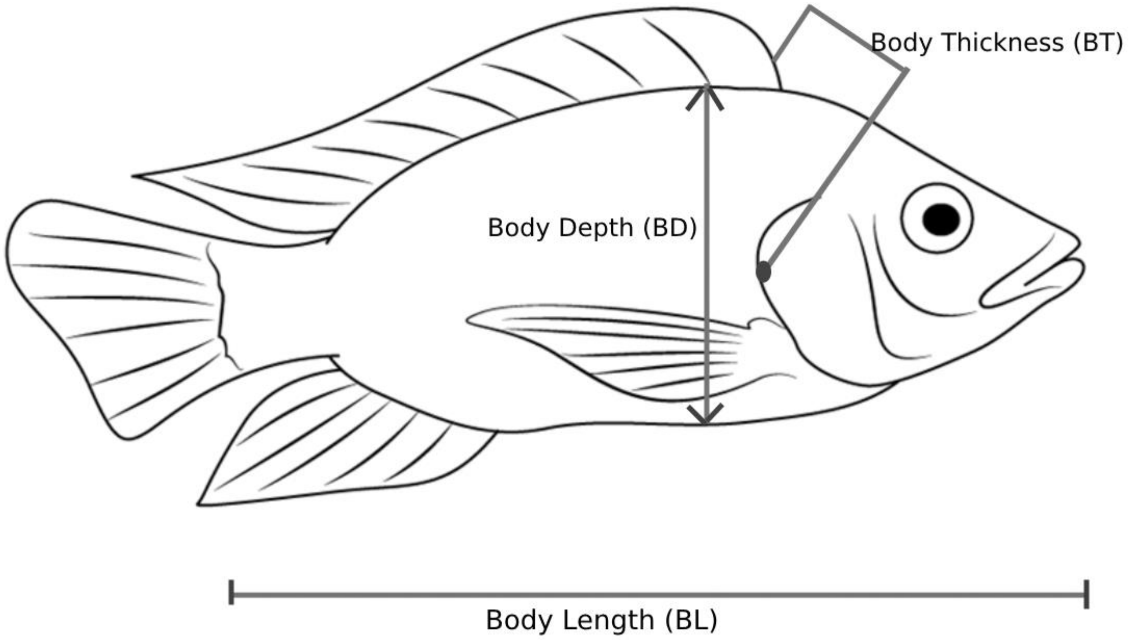

Figure S1.1: Morphometric measurements of GST®. The fish is drawn from www.drawingtutorials101.com

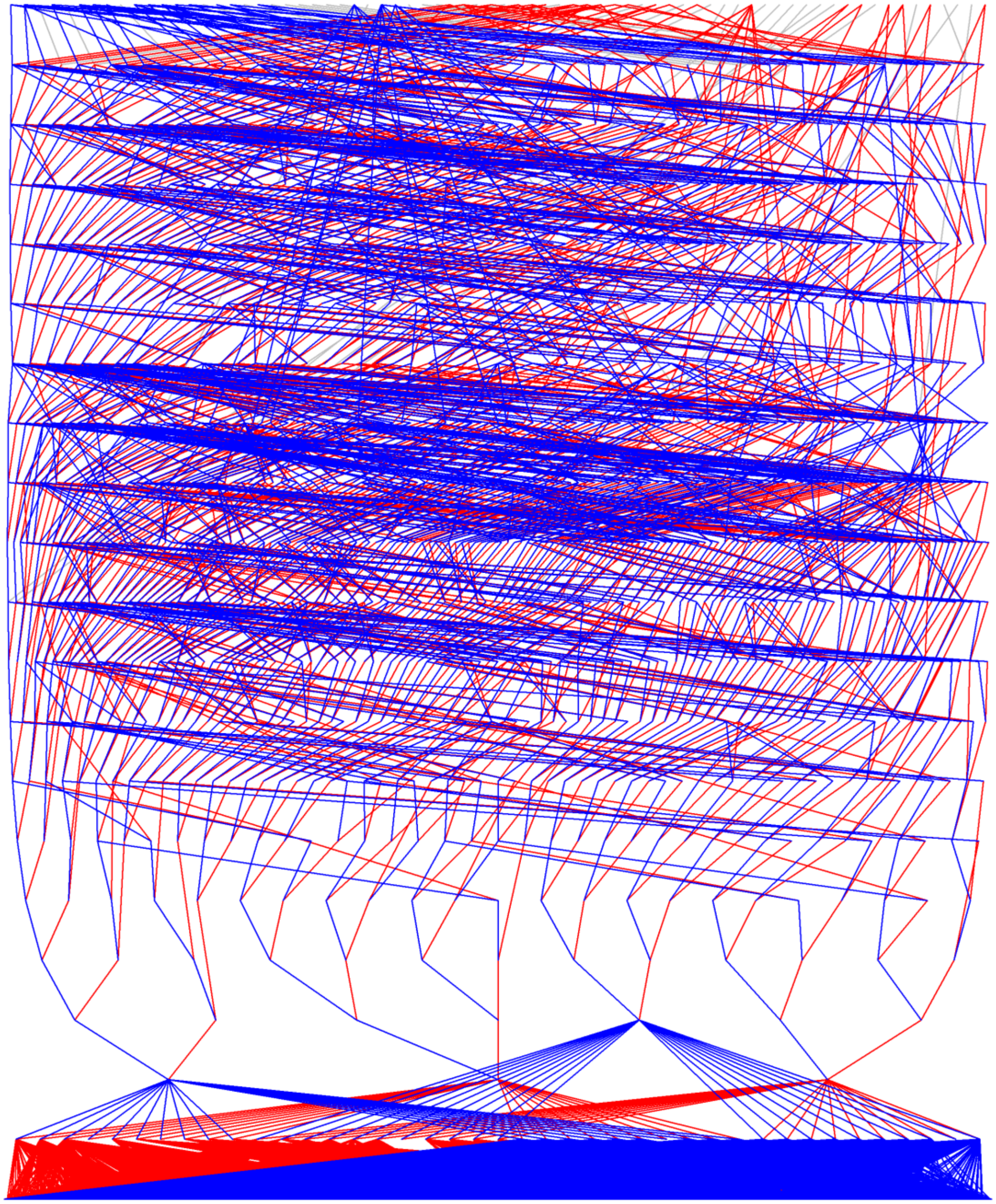

Figure S1.2: Pedigree structure of the 22 generations of GST strain generated by Pedantics package (Morrissey and Wilson, 2010)[1] in R arranged according to the depth of information available. Red, blue and grey lines represent maternal, paternal and uninformative links. The base generation is the Generation 3 of GIFT.

[1] Morrissey MB, Wilson AJ (2010). pedantics: an r package for pedigree-based genetic simulation and pedigree manipulation, characterization and viewing. *Mol Ecol Resour* **10**: 711–719.

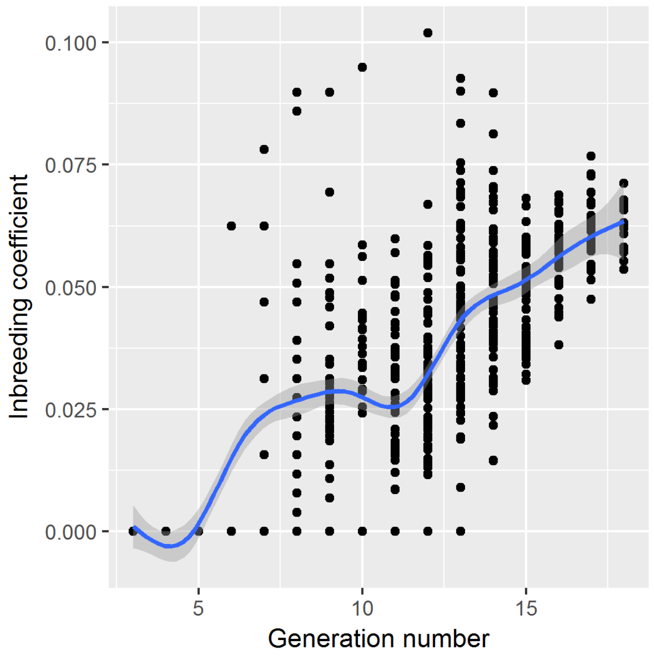

Figure S1.3: Inbreeding coefficients at different generations of pedigree for GST Tilapia. The curve was plotted using loess option in ggplot2 package (Wilkinson, 2011)[2] in R. The shaded area along the blue line represents ± one s.e.

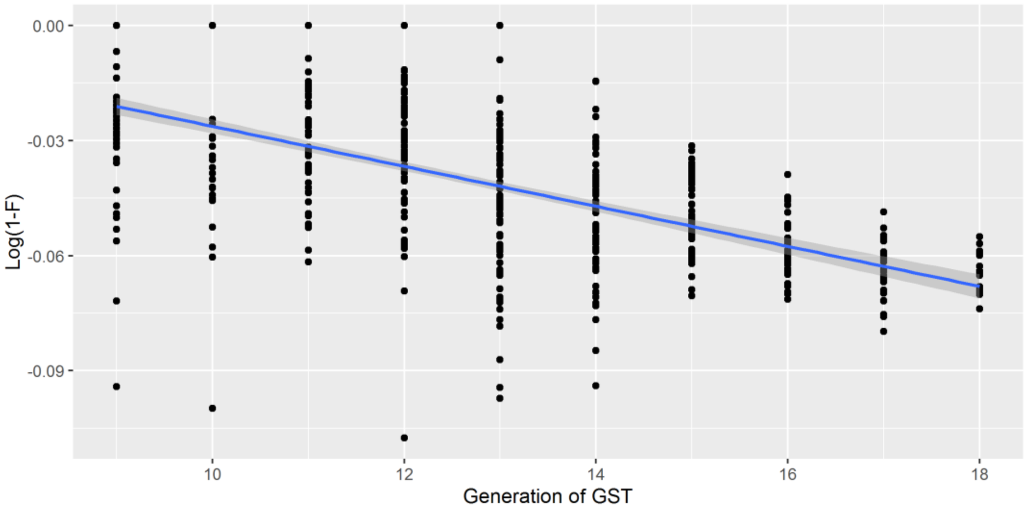

Figure S1.4: Figure showing simple regression coefficient of $\log_e(1\text{-}F)$ over the generation 9 to 18; where F is the inbreeding coefficient. The slope -0.0053 represents $-\Delta F$. N_e of the GST strain was calculated as $1/(2\Delta F) = 95$. The shady area along the blue line shows the standard error of the fitted regression line.

[2] Wilkinson L (2011). ggplot2: Elegant Graphics for Data Analysis by WICKHAM, H. *Biometrics* **67**: 678–679.

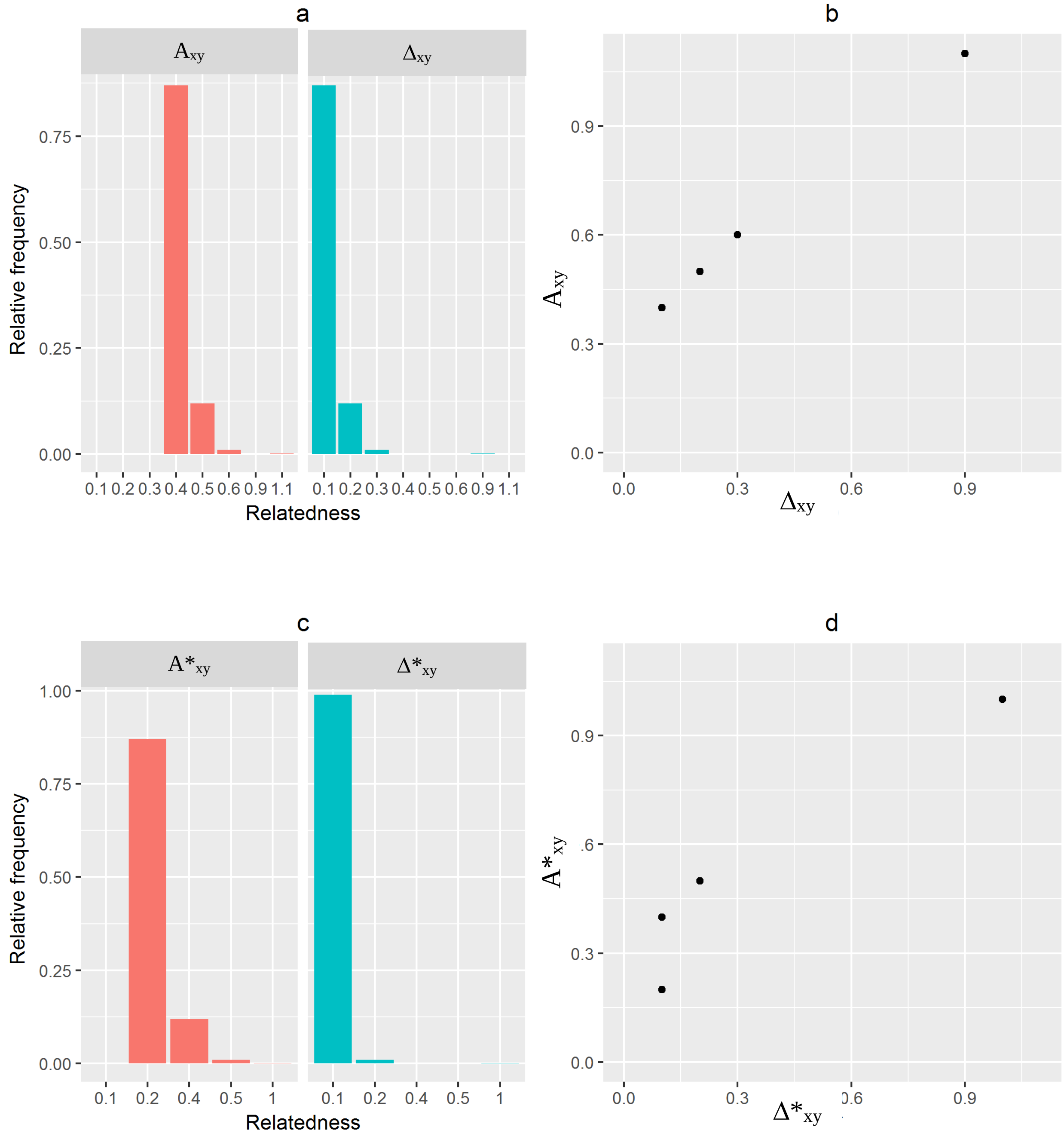

Figure S1.5: Coefficients of fraternity (Δ_{xy}) and numerator relationships (A_{xy}) between animals having phenotypic observations calculated either using the full pedigree (a and b) or only the last 3 generations (c and d) corresponding to a base generation defined by G1 to G4. Plots a and c shows the relative frequency and b and d shows the correspondence between A_{xy} and Δ_{xy}.
