# Supplementary material for: Maternal, dominance and additive genetic effects in Nile tilapia; influence on growth, fillet yield and body size traits
Source: Heredity (Edinb). 2018 Jan 16;120(5):452–62. doi: 10.1038/s41437-017-0046-x (PMC5889400; doi:10.1038/s41437-017-0046-x)
Supplement: Supplementary file 2 — Supplementary 2 Correspondence between SFM, ADM and A*D*M* models [file 41437_2017_46_MOESM2_ESM.pdf]

Supplement Information 2

Maternal, dominance and additive genetic effects in Nile tilapia; influence on growth, fillet yield and body size traits

R Joshi[1], JA Woolliams[1,2], THE Meuwissen[1] and HM Gjøen[1]

[1]Department of Animal and Aquacultural Sciences, Norwegian University of Life Sciences, 1432 Ås, Norway

[2] The Roslin Institute, Royal (Dick) School of Veterinary Studies, University of Edinburgh, Easter Bush, Midlothian, EH25 9RG, United Kingdom

Correspondence between SFM, ADM and $A^*D^*M^*$ models.

SFM MODELS

SFM models estimate the 3 core components in the factorial design directly, and these are V_{Sire}, V_{Dam}, and V_{Fsib}. Let C denote cov(y_i, y_j) for individuals *i* and *j*.

i, *j* no common parent (U), $C_U = 0$ (1)

i, *j* paternal half-sibs (PHS), $C_{PHS} = V_{Sire}$ (2)

i, *j* maternal half-sibs (MHS), $C_{MHS} = V_{Dam}$ (3)

i, *j* full-sibs (FS), $C_{FS} = V_{Sire} + V_{Dam} + V_{Fsib}$ (4)

Standard interpretation of these models (Falconer *et al.*, 1996) would imply $\sigma^2_M = C_{MHS} – C_{PHS}$, $\sigma^2_A = 4C_{PHS} = 4V_{Sire}$, and $\sigma^2_D = 4(C_{FS} – C_{PHS} - C_{MHS}) = 4V_{Fsib}$. If components $C_{MHS} < C_{PHS}$ or $C_{FS} < C_{PHS}+C_{MHS}$ further analyses may drop components and pool information, however for the current purpose these initial estimates will be used.

ADM MODELS

The covariance among different type of relationships among the phenotyped individuals relative to a base generation are described by the numerator relationships and coefficients of fraternity relative. Given the (intended) single parents selected from generation 20 these coefficients show no variation (Figure S1.2 in Supplementary 1).

For ADM models with generation 3 base and *i*, *j* in generation 22.

$$C_U = 0.357\ \sigma^2_A + 0.0928\ \sigma^2_D \quad (5)$$

$$C_{PHS} = 0.475\ \sigma^2_A + 0.1610\ \sigma^2_D \quad (6)$$

$$C_{MHS} = 0.475\ \sigma^2_A + 0.1610\ \sigma^2_D + \sigma^2_M \quad (7)$$

$$C_{FS} = 0.592\ \sigma^2_A + 0.2851\ \sigma^2_D + \sigma^2_M \quad (8)$$

Here if *i*, *j* are unrelated the covariance is the accumulated genotypic drift from the base generation and is accounted for by the mean fitted to the data in the models and equation 1 can be subtracted from the (2), (3) and (4).

$$C_{PHS} = 0.118\ \sigma^2_A + 0.069\ \sigma^2_D \quad (9)$$

$$C_{MHS} = 0.118\ \sigma^2_A + 0.069\ \sigma^2_D + \sigma^2_M \quad (10)$$

$$C_{FS} = 0.235\ \sigma^2_A + 0.193\ \sigma^2_D + \sigma^2_M \quad (11)$$

Now solving equating (9), (10) and (11) and equating them to (2) to (4).

- Estimate of σ^2_M remains unchanged as $C_{MHS} – C_{PHS}$ so moving from SFM to ADM makes no change.
- Estimate of σ^2_D is now $(C_{FS}–C_{PHS}–C_{MHS})/0.055 = 18.18(C_{FS}–C_{PHS}–C_{MHS}) =18.18V_{Fsib}$ a simple scaling of the result from the SFM model, but 4.56 times that expected from standard assumptions.
- Estimate of σ^2_A is now $19.11C_{PHS}$-$10.63(C_{FS}$-$C_{MHS}) = 8.48V_{Sire}$ -$10.63V_{Fsib}$. Therefore the estimate of σ^2_A is unaffected by the estimate of σ^2_M but reduces as the additional variance common to full-sibs increases. As a consequence a positive variance between half-sib families can yield 0 for σ^2_A if $V_{Fsib} > 0.80V_{Sire}$. If $V_{Fsib} = 0$, then σ^2_A is 2.1 -fold greater than predicted from the standard assumptions, primarily due to the sires within parent groups A and B being full-sibs, and the removal of the reciprocal cross effect as a fixed effect.

A consequence of σ^2_M and σ^2_D being estimated from the same quantity as the SMF models is that the statistical significance will be identical, and the s.e.s will have the same scaling factor as the quantity itself.

A*D*M* MODELS

For A*D*M* models with generation 20 base and *i*, *j* in generation 22.

$$C_U =0.250\ \sigma^2_A + 0.0625\ \sigma^2_D \quad (12)$$

$$C_{PHS} = 0.375\ \sigma^2_A + 0.1250\ \sigma^2_D \quad (13)$$

$$C_{MHS} = 0.375\ \sigma^2_A + 0.1250\ \sigma^2_D + \sigma^2_M \quad (14)$$

$$C_{FS} = 0.500\ \sigma^2_A + 0.2500\ \sigma^2_D + \sigma^2_M \quad (15)$$

Here if *i*, *j* are unrelated the covariance is the accumulated genotypic drift from the base generation and is accounted for by the mean fitted to the data in the models and equation 1 can be subtracted from the (2), (3) and (4).

$$C_{PHS} = 0.125\ \sigma^2_A + 0.0625\ \sigma^2_D \quad (16)$$

$$C_{MHS} = 0.125\ \sigma^2_A + 0.0625\ \sigma^2_D + \sigma^2_M \quad (17)$$

$$C_{FS} \quad = 0.250\ \sigma^2_A + 0.1875\ \sigma^2_D + \sigma^2_M \qquad (18)$$

Now solving equating (16), (17) and (18) and equating them to (2) to (4).

- Estimate of σ^2_M remains unchanged as $C_{MHS} – C_{PHS}$ so moving from SFM to A*D*M* makes no change.
- Estimate of σ^2_D is now $(C_{FS}–C_{PHS}–C_{MHS})/0.0675 = 16(C_{FS}–C_{PHS}–C_{MHS}) =16V_{Fsib}$ a simple scaling of the result from the SFM model, but 4 times that expected from standard assumptions.
- Estimate of σ^2_A is now $16C_{PHS}-8(C_{FS}-C_{MHS}) = 8(V_{Sire} - V_{Fsib})$, which is similar to the result for ADM models in structure. If $V_{Fsib} = 0$, $\sigma^2_A = 8V_{Sire}$.

Consequently the outcomes of A*D*M* will be similar to those of ADM models but with slightly different scaling factors.

EVIDENCE FROM EVALUATIONS

The EBVs from the full ADM and the full A*D*M* models were plotted for (i) the sires and (ii) all offspring and are shown in Fig. S4.1. It can be seen the EBVs from one model is a linear transformation of the other.

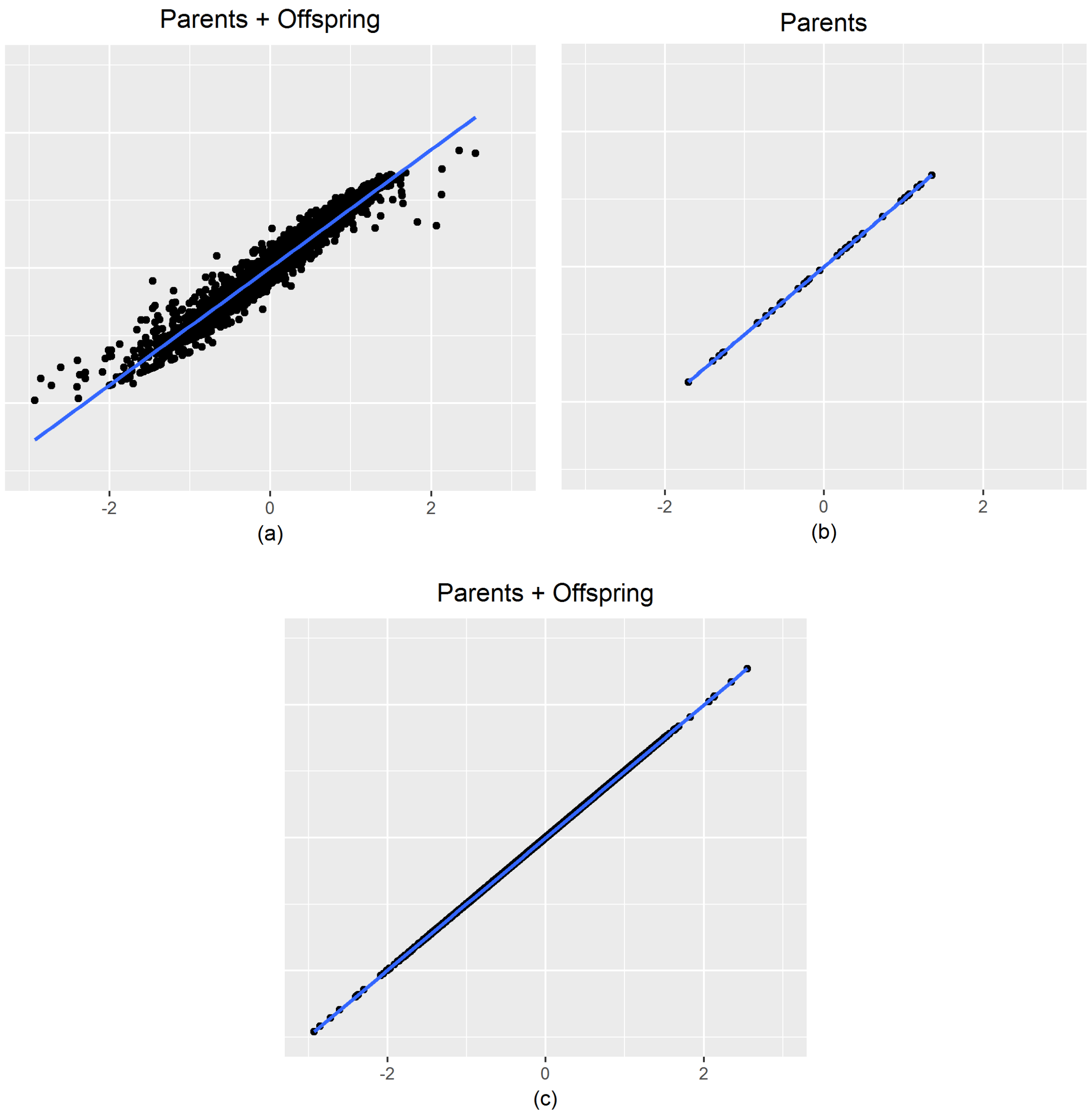

Figure S4.1: Scatterplot for the EBVs obtained from different models. Figure (a) and (b) are the comparison between A model and the model without pedigree information and figure (c) is the comparison between A and A* models.
