# Supplementary material for: Maternal, dominance and additive genetic effects in Nile tilapia; influence on growth, fillet yield and body size traits
Source: Heredity (Edinb). 2018 Jan 16;120(5):452–62. doi: 10.1038/s41437-017-0046-x (PMC5889400; doi:10.1038/s41437-017-0046-x)
Supplement: Supplementary file 3 — Supplementary 3 Tables of variance component estimates for all the models [file 41437_2017_46_MOESM3_ESM.pdf]

Supplement Information 3

Maternal, dominance and additive genetic effects in Nile tilapia; influence on growth, fillet yield and body size traits

R Joshi[1], JA Woolliams[1,2], THE Meuwissen[1] and HM Gjøen[1]

[1]Department of Animal and Aquacultural Sciences, Norwegian University of Life Sciences, 1432 Ås, Norway

[2] The Roslin Institute, Royal (Dick) School of Veterinary Studies, University of Edinburgh, Easter Bush, Midlothian, EH25 9RG, United Kingdom

Supplementary 3: Tables of variance component estimates for all the models

Table S3.1: Heritabilities for different models and different traits (standard errors are inside parenthesis). Values of 0 for component indicate estimate was bound to zero.

Trait	Model	Variance parameters					Ratios		
		Additive	Dominance	Maternal	Residual	Phenotypic	h^2	d^2	m^2
BD	A	0.26 [0.08]	-	-	0.32 [0.04]	0.58 [0.04]	0.44 [0.1]	-	-
	AD	0.15 [0.1]	0.15 [0.09]	-	0.27 [0.05]	0.57 [0.04]	0.27 [0.15]	0.26 [0.15]	-
	ADM	0 [0]	0.14 [0.05]	0.05 [0.02]	0.35 [0.04]	0.53 [0.03]	0 [0]	0.26 [0.09]	0.09 [0.04]
	AM	0.09 [0.04]	-	0.05 [0.02]	0.4 [0.02]	0.54 [0.03]	0.17 [0.07]	-	0.09 [0.04]
	A*	0.24 [0.07]	-	-	0.32 [0.04]	0.56 [0.04]	0.43 [0.1]		
	A*D*	0.16 [0.08]	0.13 [0.08]	-	0.26 [0.05]	0.55 [0.04]	0.29 [0.13]	0.24 [0.14]	
	A*D*M*	0 [0]	0.14 [0.05]	0.05 [0.02]	0.33 [0.04]	0.52 [0.03]	0 [0]	0.27 [0.09]	0.09 [0.04]
	A*M*	0.08 [0.04]	-	0.05 [0.02]	0.40 [0.02]	0.53 [0.03]	0.16 [0.07]	-	0.09 [0.04]
	S	0.12 [0.04]	-	-	0.44 [0.01]	0.47 [0.02]	0.25 [0.07]	-	
	SF	0.11 [0.04]	0.03 [0.02]	-	0.44 [0.01]	0.50 [0.02]	0.23 [0.06]	0.07 [0.04]	
	SFM	0.03 [0.02]	0.04 [0.02]	0.05 [0.02]	0.44 [0.01]	0.51 [0.03]	0.07 [0.04]	0.07 [0.04]	0.09 [0.04]
	SM	0.04 [0.02]	-	0.05 [0.02]	0.44 [0.01]	0.51 [0.03]	0.08 [0.04]	-	0.09 [0.04]

Trait	Model	Variance parameters					Ratios		
		Additive	Dominance	Maternal	Residual	Phenotypic	h^2	d^2	m^2
BL	A	0.97 [0.31]	-	-	2.33 [0.17]	3.29 [0.18]	0.29 [0.08]	-	-
	AD	0.74 [0.43]	0.33 [0.45]	-	2.2 [0.24]	3.27 [0.18]	0.23 [0.12]	0.1 [0.14]	-
	ADM	0.30 [0.4]	0.36 [0.46]	0.12 [0.09]	2.39 [0.22]	3.17 [0.15]	0.09 [0.12]	0.12 [0.14]	0.04 [0.03]
	AM	0.55 [0.26]	-	0.12 [0.09]	2.52 [0.15]	3.19 [0.15]	0.17 [0.08]	-	0.04 [0.03]
	A*	0.90 [0.29]			2.33 [0.17]	3.23 [0.16]	0.28 [0.08]		
	A*D*	0.74 [0.37]	0.28 [0.39]		2.19 [0.26]	3.21 [0.17]	0.23 [0.11]	0.09 [0.12]	
	A*D*M*	0.32 [0.34]	0.32 [0.4]	0.12 [0.09]	2.36 [0.24]	3.12 [0.15]	0.10 [0.11]	0.10 [0.13]	0.04 [0.03]
	A*M*	0.52 [0.25]		0.12 [0.09]	2.52 [0.15]	3.15 [0.14]	0.16 [0.07]		0.04 [0.03]
	S	0.45 [0.15]			2.78 [0.08]	3.00 [0.11]	0.15 [0.05]		
	SF	0.44 [0.15]	0.07 [0.1]		2.77 [0.08]	3.00 [0.11]	0.15 [0.05]	0.02 [0.03]	

Trait	Model	Variance parameters					Ratios		
	SFM	0.24 [0.12]	0.08 [0.1]	0.12 [0.09]	2.76 [0.08]	3.02 [0.12]	0.08 [0.04]	0.03 [0.03]	0.04 [0.03]
	SM	0.26 [0.12]		0.12 [0.09]	2.78 [0.08]	3.02 [0.12]	0.09 [0.04]		0.04 [0.03]

Trait	Model	Variance parameters					Ratios		
		Additive	Dominance	Maternal	Residual	Phenotypic	h^2	d^2	m^2
BT	A	1.97 [0.69]	-	-	7.44 [0.41]	9.41 [0.43]	0.21 [0.07]	-	-
	AD	0.96 [1.13]	1.51 [1.36]	-	6.86 [0.65]	9.34 [0.43]	0.10 [0.12]	0.16 [0.15]	-
	ADM	0 [0]	1.53 [0.7]	0.29 [0.2]	7.3 [0.54]	9.12 [0.35]	0 [0]	0.17 [0.07]	0.03 [0.02]
	AM	1.05 [0.56]	-	0.28 [0.2]	7.87 [0.36]	9.20 [0.38]	0.11 [0.06]	-	0.03 [0.02]
	A*	1.85 [0.65]			7.44 [0.41]	9.29 [0.39]	0.20 [0.06]		
	A*D*	1.08 [0.93]	1.32 [1.19]		6.77 [0.72]	9.17 [0.4]	0.12 [0.1]	0.14 [0.13]	
	A*D*M*	0.07 [0.84]	1.48 [1.22]	0.30 [0.2]	7.15 [0.69]	8.99 [0.36]	0.01 [0.1]	0.16 [0.14]	0.03 [0.02]
	A*M*	0.98 [0.53]		0.28 [0.2]	7.87 [0.36]	9.13 [0.36]	0.11 [0.06]		0.03 [0.02]
	S	0.92 [0.32]			8.36 [0.24]	8.83 [0.28]	0.10 [0.03]		
	SF	0.87 [0.32]	0.33 [0.3]		8.3 [0.24]	8.82 [0.28]	0.10 [0.04]	0.04 [0.03]	
	SFM	0.40 [0.26]	0.37 [0.3]	0.30 [0.2]	8.29 [0.24]	8.88 [0.31]	0.05 [0.03]	0.04 [0.03]	0.03 [0.02]
	SM	0.49 [0.26]		0.28 [0.2]	8.36 [0.24]	8.89 [0.31]	0.06 [0.03]		0.03 [0.02]

Trait	Model	Variance parameters					Ratios		
		Additive	Dominance	Maternal	Residual	Phenotypic	h^2	d^2	m^2
BWH	A	3216 [989]	-	-	4312 [496]	7528 [546]	0.43 [0.1]	-	-
	AD	2085 [1219]	1688 [1090]	-	3673 [636]	7447 [543]	0.28 [0.15]	0.23 [0.15]	-
	ADM	0 [0]	1548 [614]	637 [313]	4739 [450]	6925 [381]	0 [0]	0.22 [0.08]	0.09 [0.04]
	AM	1008 [492]	-	635 [316]	5342 [287]	6986 [405]	0.14 [0.07]	-	0.09 [0.04]
	A*	3006 [925]			4320 [495]	7326 [487]	0.41 [0.1]		
	A*D*	2149 [1069]	1475 [953]		3575 [680]	7200 [488]	0.30 [0.13]	0.20 [0.13]	
	A*D*M*	0 [0]	1557 [597]	644 [313]	4579 [494]	6780 [361]	0 [0]	0.23 [0.09]	0.10 [0.04]
	A*M*	943 [460]		636 [316]	5344 [287]	6923 [391]	0.14 [0.06]		0.09 [0.04]
	S	1503 [462]			5822 [165]	6573 [281]	0.23 [0.06]		
	SF	1443 [461]	369 [238]		5756 [165]	6570 [280]	0.22 [0.06]	0.06 [0.04]	
	SFM	378 [222]	402 [243]	746 [315]	5745 [166]	6681 [354]	0.06 [0.03]	0.06 [0.04]	0.10 [0.04]
	SM	4712 [231]		736[316]	5816 [165]	6687 [355]	0.07 [0.03]		0.10 [0.04]

Trait	Model	Variance parameters					Ratios		
		Additive	Dominance	Maternal	Residual	Phenotypic	h^2	d^2	m^2
**FW**	A	503 [155]	-	-	626 [77]	1129 [85]	0.45 [0.11]	-	-
	AD	425[180]	126 [138]	-	575 [94]	1126 [8]	0.38 [0.14]	0.11 [0.12]	-
	ADM	0 [0]	152 [71]	119[53]	755 [54]	1027 [60]	0 [0]	0.15 [0.07]	0.12 [0.05]
	AM	104 [56]	-	116 [52]	812 [36]	1033 [61]	0.10 [0.05]	-	0.11 [0.05]
	A*	470 [145]			627 [77]	1098 [76]	0.43 [0.1]		
	A*D*	413 [161]	111 [121]		568 [99]	1091 [77]	0.38 [0.12]	0.10 [0.11]	
	A*D*M*	11 [93]	142 [127]	119 [53]	743 [71]	1015 [62]	0.01 [0.09]	0.14 [0.13]	0.12 [0.05]
	A*M*	98 [53]		116 [52]	812 [36]	1026 [61]	0.10 [0.05]		0.11 [0.05]
	S	235 [73]			862 [25]	980 [43]	0.24 [0.07]		
	SF	234 [73]	28 [30]		857 [25]	981 [44]	0.24 [0.07]	0.03 [0.03]	
	SFM	41 [26]	36 [32]	120 [53]	855 [25]	1003 [58]	0.04 [0.03]	0.04 [0.03]	0.12 [0.05]
	SM	49 [26]		116 [53]	861 [24]	1002 [58]	0.05 [0.03]		0.12 [0.05]

Trait	Model	Variance parameters					Ratios		
		Additive	Dominance	Maternal	Residual	Phenotypic	h^2	d^2	m^2
**FY**	A	2.28 [0.76]	-	-	7.34 [0.44]	9.63 [0.46]	0.24 [0.07]	-	-
	AD	2.28 [0.76]	0 [0]	-	7.34 [0.44]	9.63 [0.46]	0.24 [0.07]	0 [0]	-
	ADM	2.11 [0.94]	0 [0]	0.05 [0.18]	7.42 [0.51]	9.58 [0.48]	0.22 [0.09]	0 [0]	0.01 [0.02]
	AM	2.11 [0.94]	-	0.05 [0.18]	7.42 [0.51]	9.58 [0.48]	0.22 [0.09]	-	0.01 [0.02]
	A*	2.13 [0.71]			7.35 [0.43]	9.48 [0.42]	0.23 [0.07]		
	A*D*	2.13 [0.71]	0 [0]		7.35 [0.43]	9.48 [0.42]	0.23 [0.07]	0 [0]	
	A*D*M*	1.97 [0.88]	0 [0]	0.05 [0.18]	7.43 [0.5]	9.45 [0.43]	0.21 [0.09]	0 [0]	0.01 [0.02]
	A*M*	1.97 [0.88]		0.05 [0.18]	7.43 [0.5]	9.45 [0.43]	0.21 [0.09]		0.01 [0.02]
	S	1.07 [0.36]			8.41 [0.24]	8.95 [0.29]	0.12 [0.04]		
	SF	1.07 [0.36]	0 [0]		8.41 [0.24]	8.95 [0.29]	0.12 [0.04]	0 [0]	
	SFM	0.98 [0.44]	0 [0]	0.05 [0.19]	8.41 [0.24]	8.96 [0.3]	0.11 [0.05]	0 [0]	0.01 [0.02]
	SM	0.98 [0.44]		0.05 [0.19]	8.41 [0.24]	8.96 [0.3]	0.11 [0.05]		0.01 [0.02]
