# Supplementary material for: Maternal, dominance and additive genetic effects in Nile tilapia; influence on growth, fillet yield and body size traits
Source: Heredity (Edinb). 2018 Jan 16;120(5):452–62. doi: 10.1038/s41437-017-0046-x (PMC5889400; doi:10.1038/s41437-017-0046-x)
Supplement: Supplementary file 4 — Supplementary 4 Literature reviews for heritabilities and common maternal and environmental effects in tilapia [file 41437_2017_46_MOESM4_ESM.pdf]

Supplement Information 4

Maternal, dominance and additive genetic effects in Nile tilapia; influence on growth, fillet yield and body size traits

R Joshi[1], JA Woolliams[1,2], THE Meuwissen[1] and HM Gjøen[1]

[1]Department of Animal and Aquacultural Sciences, Norwegian University of Life Sciences, 1432 Ås, Norway

[2] The Roslin Institute, Royal (Dick) School of Veterinary Studies, University of Edinburgh, Easter Bush, Midlothian, EH25 9RG, United Kingdom

Literature reviews for heritabilities and common maternal and environmental effects in tilapia.

Literature review was done for additive heritability and common maternal and environmental effect for Nile tilapia (GIFT and GST® strains). Most of the literature were found for GIFT strain and wide ranges of values were seen which might be due to different generations of study population or due to the different models used to calculate the values. Nguyen et al. (2010) have reported the common maternal and environmental effect lower than ours for some traits (BD, BWH and FW) in the GIFT strain. Despite the confounding of the maternal and common environmental effects in their model, we are not sure why our values are greater than their values.

Table S3.1: Literature reviews for heritabilities (SE are inside the parenthesis)

Traits	Heritability	Reference	Statement
**BD**	0.14 (0.037)	(Nguyen *et al.*, 2007)	Female
	0.17 (0.046)	(Nguyen *et al.*, 2007)	Male
	0.20 (0.039)	(Nguyen *et al.*, 2007)	All
	0.28 (0.17–0.41)	(Reis Neto *et al.*, 2014)	
	0.32 (0.10)	(Nguyen *et al.*, 2010)	
**BL**	0.19 (0.12)	(Rutten *et al.*, 2005)	
	0.29 (0.05)	(Nguyen *et al.*, 2007)	Female
	0.3 (0.19–0.42)	(Reis Neto *et al.*, 2014)	
	0.30 (0.05)	(Nguyen *et al.*, 2007)	All
	0.30 (0.05)	(Nguyen *et al.*, 2007)	Male
	0.31 (0.10)	(Nguyen *et al.*, 2010)	
**BT**	0.20 (0.08)	(Nguyen *et al.*, 2010)	
	0.25 (0.13)	(Rutten *et al.*, 2005)	
	0.26 (0.047)	(Nguyen *et al.*, 2007)	Female
	0.26 (0.052)	(Nguyen *et al.*, 2007)	Male
	0.29 (0.043)	(Nguyen *et al.*, 2007)	All
	0.29 (0.19 - 0.41)	(Reis Neto *et al.*, 2014)	
**BWH**	0.06 to 0.48	(Bentsen *et al.*, 2012)	Standard fertilized ponds without feed supplement
	0.17 to 0.44	(Bentsen *et al.*, 2012)	Standard fertilized pond with feed supplement
	0.26 (0.12)	(Rutten *et al.*, 2005)	
	0.31 (0.05)	(Khaw *et al.*, 2016)	log(BWH)
	0.31 (0.11)	(Nguyen *et al.*, 2010)	
	0.33 (0.05)	(Nguyen *et al.*, 2007)	Male
	0.34 (0.07)	(Ponzoni *et al.*, 2005)	
	0.35 (0.05)	(Nguyen *et al.*, 2007)	All
	0.36 (0.05)	(Nguyen *et al.*, 2007)	Female
	0.42 (0.17)	(Bentsen *et al.*, 2012)	Cage culture with feed supplement

	0.68 (0.16)<br>0.31 (0.12)	(Bentsen *et al.*, 2012)	Cage culture with commercial pellet feed
	0.33 (0.02)	(Yalew, 2007)	GST using simple model
**FW**	0.24 (0.11)	(Rutten *et al.*, 2005)	
	0.33 (0.10)	(Nguyen *et al.*, 2010)	
**FY**	0.12 (0.06)	(Rutten *et al.*, 2005)	
	0.25 (0.07)	(Nguyen *et al.*, 2010)	
	0.07 (0.03)	(Yalew, 2007)	GST using simple model

Table S3.2: Literature reviews for common maternal and environmental effects

Traits	Common Maternal and environmental effect	Reference	Statement
**BD**	0.04 (0.04)	(Nguyen *et al.*, 2010)	
	0.15 (0.02)	(Khaw *et al.*, 2012)	Cage
	0.19 (0.019)	(Nguyen *et al.*, 2007)	Female
	0.20 (0.028)	(Khaw *et al.*, 2012)	Pond
	0.24 (0.021)	(Nguyen *et al.*, 2007)	All
	0.26 (0.03)	(Nguyen *et al.*, 2007)	Male
**BL**	0.05 (0.04)	(Nguyen *et al.*, 2010)	
	0.16 (0.02)	(Nguyen *et al.*, 2007)	All
	0.16 (0.02)	(Nguyen *et al.*, 2007)	Female
	0.16 (0.02)	(Nguyen *et al.*, 2007)	Male
	0.18 (0.03)	(Khaw *et al.*, 2012)	Cage
	0.21 (0.03)	(Khaw *et al.*, 2012)	Pond
**BT**	0.05 (0.04)	(Nguyen *et al.*, 2010)	
	0.14 (0.02)	(Khaw *et al.*, 2012)	Cage
	0.15 (0.02)	(Nguyen *et al.*, 2007)	Female
	0.16 (0.02)	(Nguyen *et al.*, 2007)	All
	0.17 (0.03)	(Khaw *et al.*, 2012)	Pond
	0.18 (0.02)	(Nguyen *et al.*, 2007)	Male
**BWH**	0.08 (0.05)	(Nguyen *et al.*, 2010)	
	0.11 (0.02)	(Santos *et al.*, 2011)	By Bayesian method
	0.12 (0.02)	(Khaw *et al.*, 2016)	
	0.15 (0.03)	(Ponzoni *et al.*, 2005)	
	0.18 (0.02)	(Nguyen *et al.*, 2007)	All
	0.18 (0.02)	(Nguyen *et al.*, 2007)	Female
	0.18 (0.03)	(Khaw *et al.*, 2012)	Cage
	0.20 (0.02)	(Nguyen *et al.*, 2007)	Male
	0.26 (0.03)	(Khaw *et al.*, 2012)	Pond
**FW**	0.05 (0.04)	(Nguyen *et al.*, 2010)	
**FY**	0.00 (0.00)	(Nguyen *et al.*, 2010)	

References

1. Bentsen HB, Gjerde B, Nguyen NH, Rye M, Ponzoni RW, Palada de Vera MS, *et al.* (2012). Genetic improvement of farmed tilapias: Genetic parameters for body weight at harvest in Nile tilapia (Oreochromis niloticus) during five generations of testing in multiple environments. *Aquaculture* **338**: 56–65.
2. Khaw HL, Ponzoni RW, Hamzah A, Abu-Bakar KR, Bijma P (2012). Genotype by production environment interaction in the GIFT strain of Nile tilapia (Oreochromis niloticus). *Aquaculture* **326**: 53–60.
3. Khaw HL, Ponzoni RW, Yee HY, Aziz MA bin, Bijma P (2016). Genetic and non-genetic indirect effects for harvest weight in the GIFT strain of Nile tilapia (Oreochromis niloticus). *Aquaculture* **450**: 154–161.
4. Nguyen NH, Khaw HL, Ponzoni RW, Hamzah A, Kamaruzzaman N (2007). Can sexual dimorphism and body shape be altered in Nile tilapia (Oreochromis niloticus) by genetic means? *Aquaculture* **272**: S38–S46.
5. Nguyen NH, Ponzoni RW, Abu-Bakar KR, Hamzah A, Khaw HL, Yee HY (2010). Correlated response in fillet weight and yield to selection for increased harvest weight in genetically improved farmed tilapia (GIFT strain), Oreochromis niloticus. *Aquaculture* **305**: 1–5.
6. Ponzoni RW, Hamzah A, Tan S, Kamaruzzaman N (2005). Genetic parameters and response to selection for live weight in the GIFT strain of Nile tilapia (Oreochromis niloticus). *Aquaculture* **247**: 203–210.
7. Reis Neto RV, Oliveira CAL de, Ribeiro RP, Freitas RTF de, Allaman IB, Oliveira SN de (2014). Genetic parameters and trends of morphometric traits of GIFT tilapia under selection for weight gain. *Sci Agric* **71**: 259–265.
8. Rutten MJM, Bovenhuis H, Komen H (2005). Genetic parameters for fillet traits and body measurements in Nile tilapia (Oreochromis niloticus L.). *Aquaculture* **246**: 125–132.
9. Santos AI, Ribeiro RP, Vargas L, Mora F, Alexandre Filho L, Fornari DC, *et al.* (2011). Bayesian genetic parameters for body weight and survival of Nile tilapia farmed in Brazil. *Pesqui Agropecuária Bras* **46**: 33–43.
10. Yalew DW (2007). Genetic and phenotypic parameter estimation in selected farm tilapia population (Oreochromis niloticus). Norwegian University of Life Sciences (UMB).
